# Does closed suction drainage reduce postoperative hematoma and muscle swelling after total hip arthroplasty? A retrospective comparative study using computed tomography

**DOI:** 10.1007/s10047-026-01561-y

**Published:** 2026-06-18

**Authors:** Tomoki Asano, Keisuke Uemura, Ryo Higuchi, Sotaro Kono, Hirokazu Mae, Tetsuro Tani, Kazuma Takashima, Seiji Okada, Mitsuyoshi Yamamura, Hidetoshi Hamada

**Affiliations:** 1 Department of Orthopaedic Surgery, Osaka University Graduate School of Medicine, 2-2, Yamadaoka, Suita, Osaka 565-0871 Japan; 2 Department of Orthopaedic Medical Engineering, Osaka University Graduate School of Medicine, 2-2, Yamadaoka, Suita, Osaka 565-0871 Japan; 3 Department of Orthopaedic Surgery, Osaka Rosai Hospital, 1179-3, Nagasonecho, Kitaku, Sakai, Osaka 591-8025 Japan

**Keywords:** Computed tomography image, Drain, Hematoma, Total hip arthroplasty, Under-fascial area

## Abstract

**Purpose:**

Closed suction drainage has been used after total hip arthroplasty (THA) to prevent hematoma, muscle swelling, and complications, but its effectiveness remains controversial. This study evaluated the effects of drains on postoperative hematoma, muscle swelling, and clinical outcomes.

**Methods:**

A total of 168 patients who underwent unilateral cementless THA via the posterior approach for secondary hip osteoarthritis due to developmental dysplasia were retrospectively reviewed. Among them, 60 matched pairs, with and without drains, were selected using propensity score matching based on age, sex, body mass index, operative time, and femoral stem type. The primary outcome was the percentage increase in under-fascial area (UFA) measured on axial computed tomography at the teardrop level. UFA was used as a surrogate marker of postoperative hematoma and muscle swelling and was assessed preoperatively and on postoperative day 7. Secondary outcomes included postoperative estimated blood loss (PEBL), pain (assessed using the numeric rating scale), and laboratory parameters (albumin, hemoglobin, white blood cell count, creatine kinase, and C-reactive protein) on postoperative days 1, 4, 7, and 14.

**Results:**

The mean percentage increase in the UFA did not differ between groups (10.7% [no-drain] vs. 11.2% [drain], *P* = 0.63). No significant differences were found in the PEBL, numeric rating scale pain scores on day 3, or laboratory parameters beyond postoperative day 14.

**Conclusion:**

Closed suction drainage did not reduce the UFA increase or improve clinical outcomes after THA. The routine use of drains may not be warranted considering the absence of demonstrable benefits and potential risks.

## Introduction

 Since its introduction by Waugh et al. in 1961, closed suction drainage has traditionally been used in total hip arthroplasty (THA) to reduce the formation of postoperative hematomas and prevent related complications (e.g., infection and increased wound tension) [1, 2]. However, recent studies have reported no remarkable reduction in hematoma formation or improvement in clinical outcomes with its use [3–5]. In addition, there are concerns regarding retrograde infection, increased workload because of drain care, patient discomfort, and delayed mobilization [6], leading to a recent trend toward omitting drains. Nonetheless, there is no clear consensus regarding the necessity of drains in THA, leaving the use of drains to each institution’s or surgeon’s preferences.

Currently, most previous studies analyzed indirect clinical parameters (e.g., estimated blood loss, transfusion requirements, or infection rates) to determine the effectiveness of drains [7, 8]. By contrast, few studies used ultrasonographic measurements of the anterior joint space in the supine position to directly assess hematoma size [9, 10]. Although these studies using ultrasound provided important information regarding the effectiveness of drains, such assessments have technical difficulty in assessing intramuscular and posterior region hematoma, leaving uncertainty regarding the total hematoma formation around the hip joint. Furthermore, ultrasonography-based measurements are subject to considerable variability because of examiner-dependent factors, including probe orientation, positioning, and contact pressure [11, 12].

To this end, we proposed to use preoperative and postoperative computed tomography (CT) to objectively assess postoperative soft-tissue changes associated with hematoma and muscle swelling around the hip joint and (1) determine whether omitting closed suction drainage is noninferior to drainage in terms of postoperative hematoma formation and muscle swelling after THA and (2) assess early postoperative outcomes following THA.

## Materials and methods

### Ethical approval

This retrospective study received approval from our institution’s Institutional Review Board (IRB number: 21115). It was conducted in accordance with the Declaration of Helsinki and its subsequent amendments, or similar ethical guidelines. Informed consent was obtained concerning the study content in an opt-out fashion.

### Participants

**Fig. 1:**
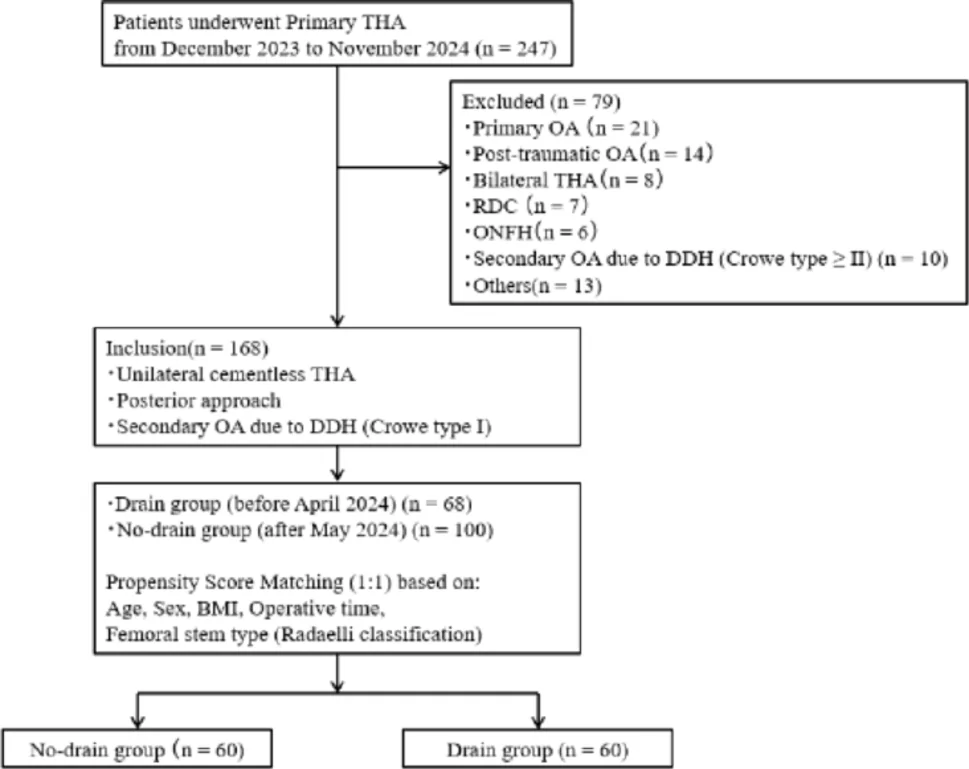
Flowchart illustrating the selection and allocation of patients for each group. Abbreviations: THA, total hip arthroplasty; OA, osteoarthritis; RDC, rapidly destructive coxarthrosis; ONFH, osteonecrosis of the femoral head; DDH, developmental dysplasia of the hip; BMI, body mass index.

The initial participants of this study included 247 patients who underwent primary THA at a single institution between December 2023 and November 2024 (Fig. 1). Of these, 168 patients met the following inclusion criteria: unilateral, cementless THA via a posterior approach for secondary hip osteoarthritis caused by developmental dysplasia (Crowe group I hip [13]). Among them, 68 patients underwent THA with a drain before May 2024, and 100 patients underwent THA without a drain thereafter. The patients were matched 1:1 using propensity score matching based on age, sex, body mass index (BMI), operative time, and femoral stem type according to the Radaelli classification [14], which categorizes stem designs as flat taper (type A), quadrangular taper (type B), fit-and-fill (type C), and calcar-guided short stems (type F). After matching, 60 patient pairs were identified and compared in the no-drain and drain groups (Fig. 1). No statistically significant differences were observed in baseline characteristics between the two groups, including age, sex, BMI, operative time, femoral stem type, medication-treated comorbidities such as hypertension and diabetes, and the use of anticoagulant therapy (Table 1).

**Table 1:** Demographic data

Variables	No-drain (*n* = 60)	Drain (*n* = 60)	*P*-value
Age (years)^a^	71.7 ± 9.7	70.3 ± 9.2	0.41
Gender^b^, n (%)			1.00
Male	7 (11.7)	7 (11.7)	
Female	53 (88.3)	53 (88.3)	
BMI (kg/m^2^)^a^	24.4 ± 5.5	24.2 ± 3.9	0.80
Operative time (min)^a^	71.1 ± 11.0	71.1 ± 11.8	1.00
Stem type (Radaelli classification)^b^, n (%)			0.99
A	14 (23.3)	16 (26.7)	
B	15 (25.0)	14 (23.3)	
C	21 (35.0)	20 (33.3)	
F	10 (16.7)	10 (16.7)	
Anticoagulant therapy^b^, n (%)	3 (5.0)	2 (3.3)	1.00
Comorbidities^b, c^, n (%)	35 (58.3)	27 (45.0)	0.20

BMI, body mass index

^a^Data are expressed as the mean ± standard deviation

^b^Data are expressed as the number of cases (percentage of column header population)

^c^Comorbidities include medication-treated hypertension and diabetes, as well as use of anticoagulant therapy

### Surgical procedure

All THA procedures were performed by a board-certified orthopaedic surgeon under general anesthesia using a posterior approach. The surgery was assisted by a CT-based hip navigation system (NAV3i Platform, Stryker Orthopaedics, Mahwah, NJ, USA) and cementless femoral stems and acetabular cups were used in all cases. Tranexamic acid (1 g) was administered 15 min before skin incision and immediately before wound closure. An intraoperative cell salvage system was used, and the collected blood was reinfused before wound closure. In the drain group, the drain was removed on the morning of postoperative day 1. Edoxaban (15 mg) was administered for seven days starting on postoperative day 1 to prevent deep vein thrombosis, except in patients already receiving anticoagulant or antithrombotic medications.

### CT imaging and postoperative evaluation of hematoma and muscle swelling

The preoperative CT scans for CT-based navigation (acquired one month before THA) and postoperative CT scans (acquired seven days after THA) to confirm the accuracy of implant placement to enhance range of motion rehabilitation were analyzed herein. Using the same protocol, preoperative and postoperative CT images were acquired with SOMATOM Definition Edge (Siemens Healthineers, Erlangen, Germany) with a slice interval of 2 mm.

**Fig. 2:**
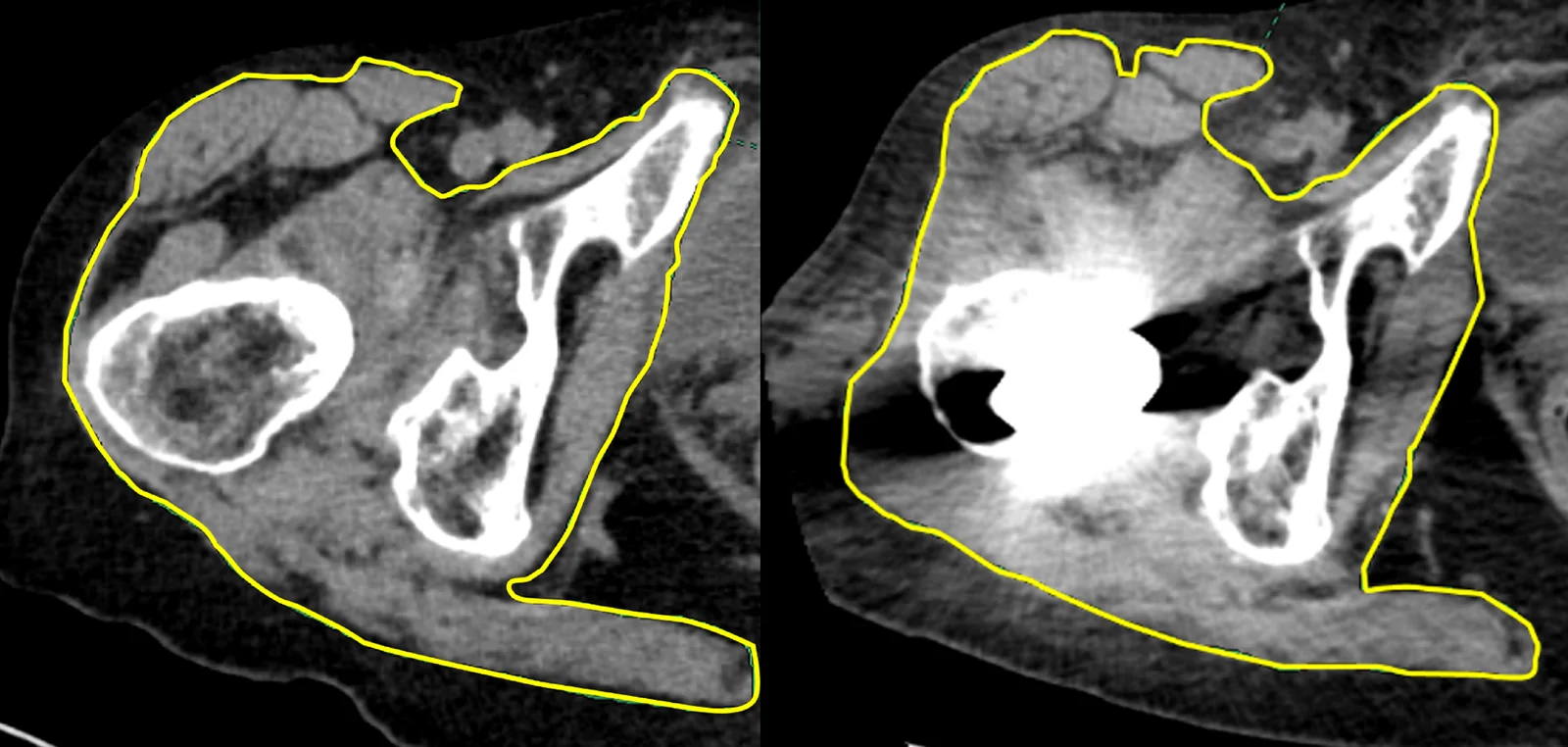
Preoperative (left) and postoperative (right) axial computed tomography images at the teardrop level, showing the under-fascial area outlined in yellow

Because metal artifacts in postoperative CT images hinder direct measurement of hematoma, we adopted a method that evaluates the increase in under-fascial area (UFA) as a surrogate marker of hematoma formation and muscle swelling. Specifically, UFA was measured on axial CT images acquired preoperatively and postoperatively at the same anatomical level to allow comparison of postoperative changes reflecting hematoma formation and muscle swelling, which may affect wound healing and activities of daily living [15]. UFA was assessed using both unadjusted values and values normalized by height squared, in accordance with a previously reported method for muscle mass normalization [16]. As the UFA is located close to the hip rotation center, it was measured at the teardrop (an anatomical landmark that does not change postoperatively) level [17]. UFA measurement was performed using the SYNAPSE VINCENT 3D Image Analysis system (Fujifilm Co., Tokyo, Japan) (Fig. 2). The percentage change in the UFA from preoperative CT to postoperative CT was calculated and compared between the no-drain and drain groups. Based on the hypothesis that closed suction drainage does not have a clinically meaningful effect on postoperative UFA increase after THA, noninferiority of the no-drain group compared with the drain group with respect to UFA increase was evaluated as the primary outcome.

### Assessment of postoperative clinical and laboratory parameters

**Fig. 3:**
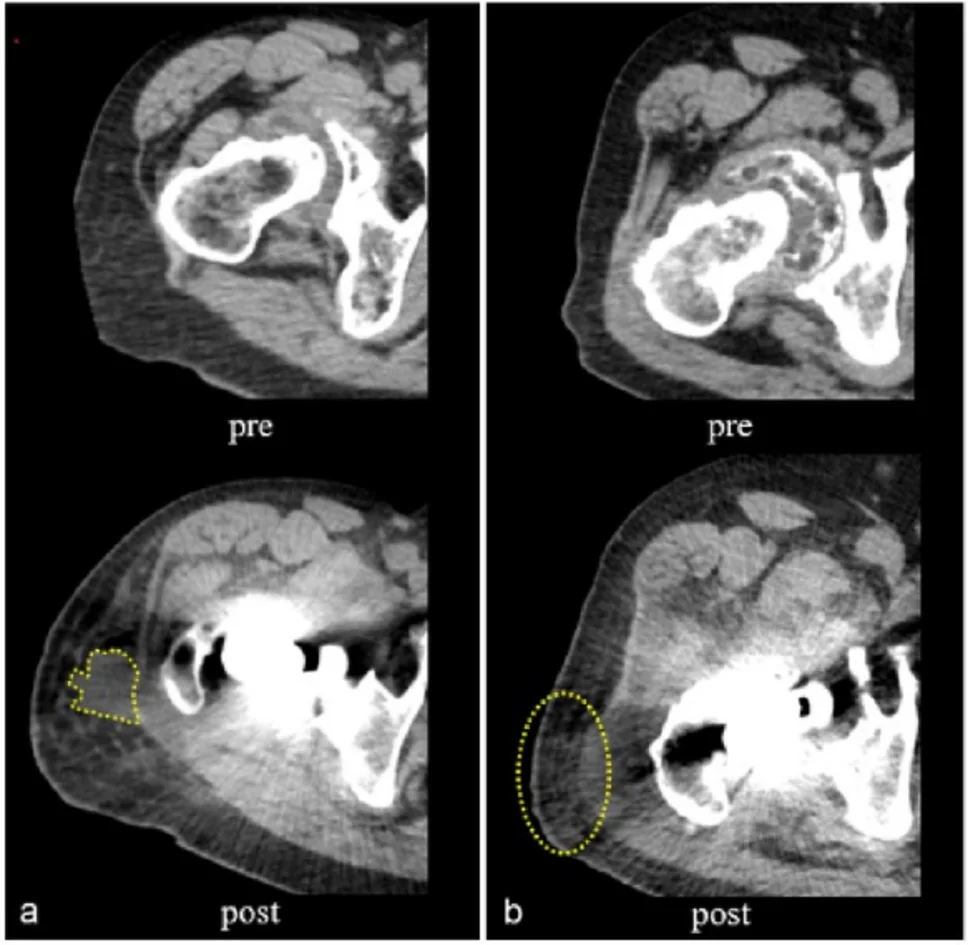
Two representative cases with and without subcutaneous hematoma. (**a**) Case with subcutaneous hematoma, outlined by a yellow dashed line. (**b**) Case without subcutaneous hematoma; the expected location is outlined by a yellow dashed line. Preoperative and postoperative computed tomography scans are shown

The pain intensity was assessed using the numeric rating scale (NRS) on postoperative day 3 to assess the effects of drains on postoperative outcomes. Moreover, the presence of subcutaneous hematoma was evaluated using CT. Specifically, cases with a soft tissue mass outside the fascia were classified as having a subcutaneous hematoma, whereas these findings without were classified as not having a subcutaneous hematoma (Fig. 3). In addition, laboratory parameters, including serum albumin, creatine kinase (CK), C-reactive protein (CRP), white blood cell count, and hemoglobin (Hb), were measured preoperatively and postoperatively on days 1, 4, 7, and 14, except for seven patients who did not have blood tests on postoperative day 14. These parameters were determined in accordance with previous studies [7, 18]. The postoperative estimated blood loss (PEBL) was calculated by subtracting intraoperative blood loss from the total blood loss, which was estimated using Nadler’s formula for patient blood volume [19] and Good’s formula for blood loss estimation based on the lowest postoperative Hb value recorded during the observation period [20, 21].

### Reproducibility of image measurements

To assess the intraobserver and interobserver reproducibility of UFA measurements on CT images, the intraclass correlation coefficients (ICCs) were calculated. Specifically, for intraobserver reliability, one board-certified orthopaedic surgeon measured 20 randomly selected cases twice, with a 6-month interval between measurements. For interobserver reliability, another board-certified orthopaedic surgeon independently measured the same 20 cases. The ICC was calculated using a two-way random-effects model for absolute agreement (ICC [1, 2]).

### Data analysis

The Shapiro–Wilk test was used to assess the normality of data distributions. Continuous variables are presented as the mean ± standard deviation. Intergroup comparisons were performed using Student’s *t*-test for continuous variables and Fisher’s exact test for categorical variables, including those with more than two categories. The NRS pain score was treated as an ordinal variable and is presented as the median (interquartile range). Intergroup comparisons of the NRS pain score were performed using the Mann–Whitney U test.

To confirm the sample size, a power analysis was performed under the assumption of noninferiority. Specifically, the noninferiority margin was set at 3% based on a previous study [22]. Assuming a power of 90% and a two-sided significance level of 0.05, the required sample size was calculated to be 47 patients per group.

All statistical analyses were performed using R software (version 4.5.0; R Foundation for Statistical Computing, Vienna, Austria). Statistical significance was considered at *P* < 0.05.

## Results

### Evaluation of postoperative hematoma and muscle swelling

No significant differences were observed between the no-drain and drain groups in either unadjusted or height-adjusted UFA, both preoperatively and postoperatively (Table 2). The mean percentage increase in the UFA was 10.7 and 11.2% in the no-drain and drain groups, respectively, and no statistically significant difference was observed between groups (95% confidence interval [CI], − 1.5 to 2.6; *P* = 0.63) (Table 2).

**Table 2:** Results of the UFA

Parameters	No-drain (*n* = 60)	Drain (*n* = 60)	*P*-value
Preoperative UFA (cm^2^)^a^	139.2 (105.5 to 218.9)	138.0 (100.4 to 224.4)	0.78
Postoperative UFA (cm^2^)^a^	154.2 (113.2 to 235.7)	153.1 (108.3 to 226.7)	0.81
Height-adjusted preoperative UFA (cm^2^/m^2^)^a^	58.6 (45.5 to 78.0)	57.8 (39.7 to 76.1)	0.59
Height-adjusted postoperative UFA (cm^2^/m^2^)^a^	64.8 (49.6 to 85.5)	64.1 (42.9 to 82.9)	0.68
%ΔUFA^a^	10.7 (0.2 to 28.2)	11.2 (1.0 to 24.0)	0.63

UFA, under-fascial area; height-adjusted UFA, UFA divided by height squared; %ΔUFA, percentage increase in the UFA from preoperative to postoperative measurements.

^a^Data are expressed as the mean (range).

### Assessment of postoperative clinical and laboratory parameters

No significant differences were observed in the PEBL (*P* = 0.15), pain intensity on postoperative day 3 as measured by the NRS (*P* = 0.59), or incidence of subcutaneous hematoma (*P* = 0.58) between the no-drain and drain groups (Table 3).

**Table 3:** Postoperative clinical parameters

Parameters	No-drain (*n* = 60)	Drain (*n* = 60)	*P*-value
PEBL (mL)^a^	486.2 (78.0 to 995.5)	563.6 (51.1 to 1961.5)	0.15
NRS^b^	3 (2 to 5)	3 (1 to 4)	0.59
Subhematoma^c^, n (%)	33 (55)	29 (48)	0.58

PEBL, postoperative estimated blood loss; Subhematoma, presence of subcutaneous hematoma; NRS, postoperative pain assessed by the numeric rating scale (NRS) on postoperative day 3

^a^Data are expressed as the mean (range)

^b^Data are expressed as the median (interquartile range)

^c^Data are expressed as the number of cases (percentage of column header population)

**Fig. 4:**
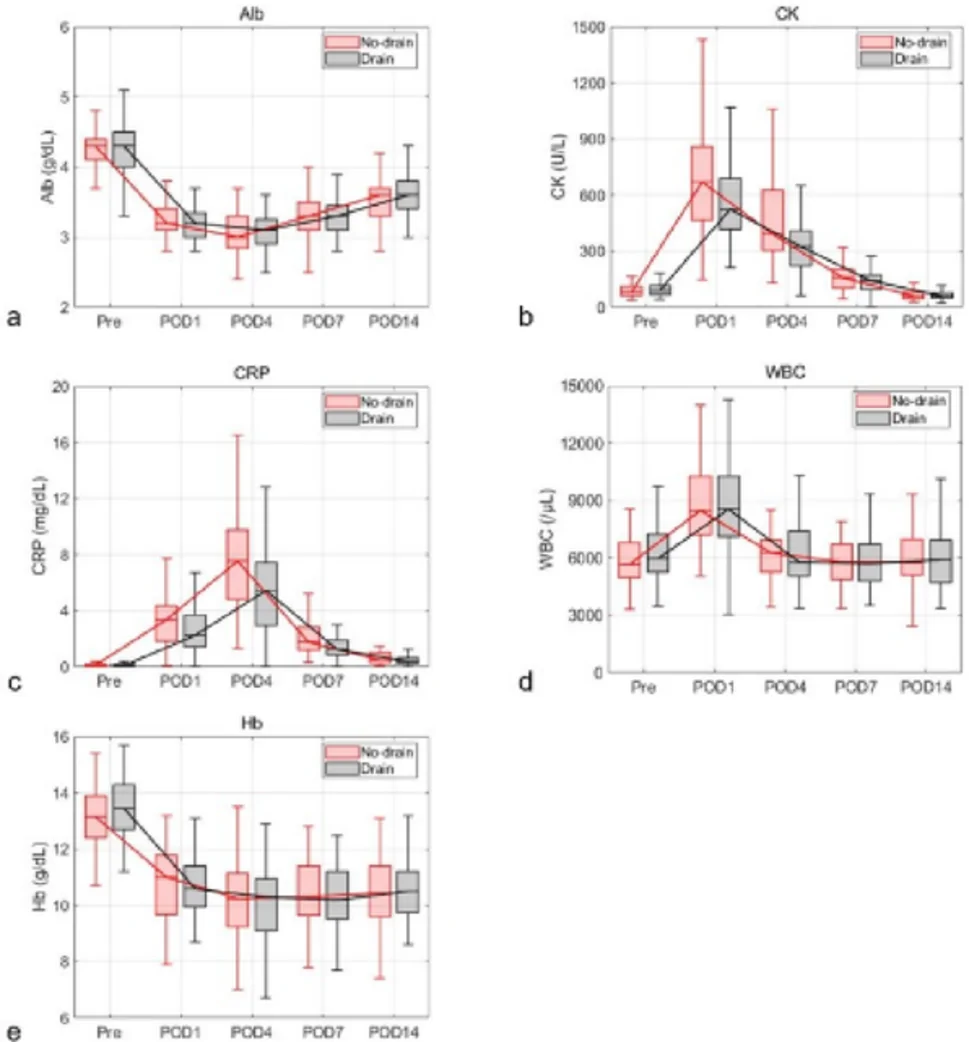
Serial changes in blood test results on postoperative days 1, 4, 7, and 14 for (**a**) albumin (Alb), (**b**) creatine kinase (CK), (**c**) C-reactive protein (CRP), (**d**) white blood cell count (WBC), and (**e**) hemoglobin (Hb)

With regard to laboratory parameters, CK and CRP levels on postoperative day 4 were significantly higher in the no-drain group than in the drain group (CK: 498.2 vs. 345.8 IU/L, *P* = 0.002; CRP: 7.82 vs. 5.98 mg/dL, *P* = 0.01). In addition, CRP levels on postoperative day 7 were significantly higher in the no-drain group than in the drain group (2.48 vs. 1.67 mg/dL, *P* = 0.02). However, no significant differences in any laboratory parameters were detected by postoperative day 14 (Fig. 4). No surgical site infections were observed in either group within 90 days postoperatively. Further, there were no differences between the groups in the timing of postoperative mobilization or in other postoperative complications (Table 3).

### Reproducibility of image measurements

The ICC for UFA measurements demonstrated excellent reproducibility, with intraobserver and interobserver ICCs of 0.98 (95% CI: 0.94–0.99, *P* < 0.001) and 0.96 (95% CI: 0.91–0.99, *P* < 0.001), respectively.

## Discussion

This study used CT-based UFA measurements to evaluate the noninferiority of no closed suction drainage compared with drainage in terms of postoperative UFA increase and clinical outcomes. We found no significant differences in postoperative UFA changes or clinical outcomes between the no-drain and drain groups after primary cementless THA, suggesting that closed suction drainage has limited clinical effectiveness in preventing postoperative hematoma accumulation and muscle swelling.

### Evaluation of postoperative hematoma

Previous studies have reported inconsistent findings regarding the effectiveness of closed suction drainage after THA. Although some studies suggested that drains help reduce hematoma formation [4, 10], others found no significant differences in hematoma size between patients with and without drains [7, 9]. The present results are consistent with those of the latter, indicating no statistically significant difference in the increase in UFA between the two groups. In addition, when the noninferiority margin was set at 3% based on a previous study [22], the 95% confidence interval (− 1.5 to 2.6) for the between-group difference in the percentage increase in UFA was within the predefined margin, suggesting that the no-drain group was noninferior to the drain group in terms of postoperative UFA increase. However, considering that our study differs from previous studies in that it used CT imaging and propensity score matching to balance multiple factors between groups, direct comparison should be made with caution.

### Assessment of postoperative clinical and laboratory parameters

No clinically meaningful differences were observed between the no-drain and drain groups in terms of PEBL, pain scores, presence of subcutaneous hematoma, or laboratory parameters. With regard to blood loss, previous studies have reported conflicting findings, with some suggesting increased bleeding with drain use [23, 24] and others finding no significant differences between groups [4, 7, 25]. Our results are consistent with those of the latter, showing no significant difference in estimated postoperative blood loss. The postoperative pain scores were also comparable between groups, which aligns with earlier studies reporting no association between drainage and pain reduction [7, 26, 27]. Similarly, the presence of subcutaneous hematoma did not significantly differ between groups, which is consistent with previous findings [9].

With regard to laboratory data, no significant differences in Hb trends were observed, consistent with earlier reports [5]. However, CK and CRP levels on postoperative day 4 and CRP levels on postoperative day 7 were higher in the no-drain group, suggesting greater inflammatory responses around the peak period. However, these differences had resolved by day 14, indicating limited clinical relevance.

### Reproducibility of image measurements

The postoperative hematoma size has traditionally been assessed using ultrasonography, with most previous studies measuring hematoma thickness from the anterior aspect of the hip joint in the supine position [9, 10]. However, in the supine position, hematomas are likely to accumulate in the posterior or posterolateral regions because of gravitational effects. This raises concerns about whether anterior ultrasound assessments accurately reflect the true hematoma size. By contrast, CT enables the quantitative evaluation of the UFA across multiple regions, including the anterior and posterior compartments and intramuscular regions, enabling a more comprehensive assessment of the UFA, which reflects hematoma formation and muscle swelling.

Ultrasound and CT measurements cannot be directly compared. However, according to previous studies on the quadriceps muscle thickness, CT-based measurements provide higher precision compared with ultrasonography [28], and the intraobserver and interobserver ICCs for ultrasonographic measurements of quadriceps muscle thickness reportedly range from 0.74 to 0.83 and from 0.76 to 0.83, respectively, indicating limited reliability [29]. By contrast, our study demonstrated excellent reproducibility of CT-based UFA measurements, with intraobserver and interobserver ICCs of 0.98 and 0.96, respectively, thereby supporting the high reliability of the results found herein.

### Clinical relevance

The primary rationale for drain placement is to reduce hematoma accumulation, aiming to minimize the risks of infection and delayed wound healing [1, 29]. However, the present study demonstrated that drain placement after THA has limited clinical effectiveness. Discontinuing the use of drains may reduce the workload of residents and nursing staff and contribute to a reduction in healthcare costs [30]. Moreover, it may relieve patients of psychological stress because of mobility restrictions and physical stress associated with additional procedures. These advantages demonstrate the clinical significance of discontinuing drains in terms of optimizing the use of limited medical resources and reducing patient burden.

### Limitations

This study has some limitations. The evaluation of the UFA using CT was performed only at a single axial slice at the teardrop level. Therefore, whether the results would differ if the UFA was evaluated at other levels remains unclear. However, the teardrop level is close to the hip rotation center [17] and does not change after THA. Thus, the UFA measured at this level likely reflects overall hematoma burden around the hip joint. In addition, although several factors were matched between groups, there may be other factors that may affect the outcomes. For example, although the Radaelli classification was matched between groups, other implant-related factors, including stem size, may affect the results. However, each stem was selected based on preoperative 3D planning to ensure proper canal fit. Thus, the impact of stem size on the study outcomes is likely to be small.

## Conclusion

Using CT-based UFA as a surrogate for hematoma and muscle swelling, this study demonstrated the noninferiority of no closed suction drainage compared with drainage in terms of postoperative UFA increase and clinical outcomes after primary cementless THA. Considering the objective and highly reproducible assessment enabled by CT, our results indicate that the routine use of drains in this surgical setting may be unnecessary.
